# Performance and Impact on Antibiotic Prescriptions of a Multiplex PCR in a Real-Life Cohort of Critically Ill Patients with Suspected Ventilated Pneumonia: A Retrospective Monocentric Observational Study

**DOI:** 10.3390/antibiotics12121646

**Published:** 2023-11-21

**Authors:** Emma Chambe, Perrine Bortolotti, Rémy Diesnis, Caroline Laurans, Rozenn Héquette-Ruz, Sophie Panaget, Patrick Herbecq, Anne Vachée, Agnès Meybeck

**Affiliations:** 1 Department of Critical Care, Victor Provo Hospital, 59100 Roubaix, France; e.chambe33@gmail.com (E.C.); perrine.bortolotti@ch-roubaix.fr (P.B.); patrick.herbecq@ch-roubaix.fr (P.H.); 2 Infectious Risk Management Unit, Victor Provo Hospital, 59100 Roubaix, France; caroline.laurans@ch-roubaix.fr (C.L.); rozenn.hequette@ch-roubaix.fr (R.H.-R.); sophie.panaget@ch-roubaix.fr (S.P.); 3 Department of Biostatistics, Victor Provo Hospital, 59100 Roubaix, France; remy.diesnis@ch-roubaix.fr; 4 Department of Microbiology, Victor Provo Hospital, 59100 Roubaix, France; anne.vachee@ch-roubaix.fr; 5 University Department of Infectious Diseases, Centre Hospitalier Dron Hospital, 59200 Tourcoing, France

**Keywords:** pneumonia, multiplex PCR, COVID-19, antibiotic use, intensive care

## Abstract

Pulmonary multiplex polymerase chain reaction (m-PCR) allows rapid pathogen detection. We aimed to assess its impact on initial antibiotic prescriptions in ventilated patients with suspected pneumonia. Between November 2020 and March 2022,ventilated patients with suspected pneumonia hospitalized in our ICU who benefited from respiratory sampling simultaneously tested using conventional microbiological methods and m-PCR were included. The proportion of appropriate changes in the initial antibiotic therapy following m-PCR results was assessed. We analyzed 104 clinical samples. Of the 47 negative m-PCR results, 16 (34%) led to an appropriate antibiotic strategy: 8 cessationsand 8 lack of initiation. Of the 57 positive m-PCR results, 51 (89%) resulted in an appropriate antibiotic strategy: 33 initiations, 2 optimizations, and 9 de-escalations. In the multivariate analysis, a positive m-PCR was associated with an appropriate antibiotic change (OR: 96.60; IC95% [9.72; 960.20], *p* < 0.001). A higher SAPS II score was negatively associated with an appropriate antibiotic change (OR: 0.96; IC95% [0.931; 0.997], *p* = 0.034). In our cohort, a positive m-PCR allowed for early initiation or adjustment of antibiotic therapy in almost 90% of cases. A negative m-PCR spared antibiotic use in onethird of cases. The impact of m-PCR results was reduced in the most severe patients.

## 1. Introduction

Ventilator-associated pneumonia (VAP)is among the most frequent infections in intensive care units (ICUs) [1]. They induce significant morbidity and mortality [2,3]. The COVID-19 pandemic resulted in a high incidence of Acute Respiratory Distress Syndrome (ARDS), requiring ventilation support and leading to high mortality [4,5]. Patients with SARS-CoV-2 infection complicated with ARDS are at high risk of bacterial pulmonary coinfection or superinfection [6,7,8]. A prolonged length of sedation and mechanical ventilation, relative immunosuppression induced by a viral infection, and corticosteroid prescription are all factors favoring the occurrence of VAP [9,10,11].

In the case of VAP, early prescription of an appropriate antimicrobial therapy could reduce mortality [12]. Rapid recognition of VAP is, therefore, essential to improving prognosis. Traditionally, diagnosis of VAP is based on clinical suspicion, new or progressive radiographic infiltrates, and microbiological diagnosis, meaning positive microbiological cultures from the lower respiratory tract [13]. However, in ICU patients, clinical signs such as fever or hypoxemia are not specific toVAP, and discovering new radiologic infiltrates is random. In patients with severe COVID-19, the diagnosis of VAP is even more challenging since substantial overlap exists between the basic clinical symptoms and signs of COVID-19 with secondary infections [14,15]. This confusion leads to frequent initiation of empirical large-spectrum antimicrobial therapy while waiting for microbiological results. A challenge in diagnosing pulmonary coinfection orsuperinfection in critically ill patients with COVID-19 is reducing the time from sampling to pathogen identification. Pulmonary multiplex polymerase chain reaction (m-PCR) led to effective and rapid pathogen detections and could help clinicians choose targeted antimicrobial therapy. Its performance, as well as its use in nosocomial pulmonary infections in the ICU, were evaluated in combination with expert opinion [16]. This study demonstrated the theoretical potential of m-PCR in reducing unnecessary antibiotic treatment, comparing hypothetical m-PCR-driven antibiotic prescriptions by experts to culture-driven antibiotic prescriptions by clinicians unaware of m-PCR results. However, data on m-PCR real-life use remains limited, especially in the context of the COVID-19 pandemic. Its use was evaluated in COVID-19 patients suspected of community-acquired pneumonia (CAP), hospital-acquired pneumonia (HAP), or VAP, showing a lower proportion of de-escalation compared with those observed in studies simulating the impact of m-PCR [17]. We conducted a retrospective monocentric study to assess the impact of initial antibiotic prescription of m-PCR in ventilated patients with suspected pneumonia.

## 2. Results

### 2.1. Demographic and Clinical Data

**Figure 1:**
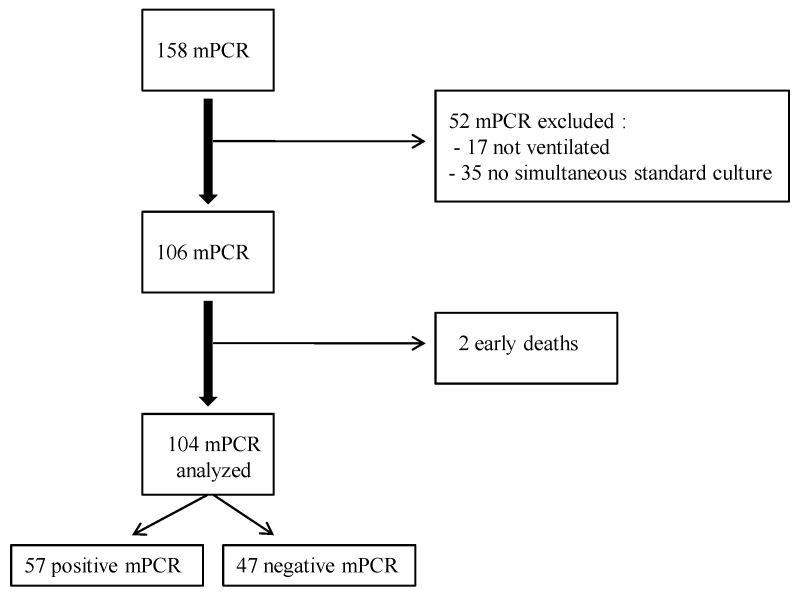
Study flowchart.

During the study period, 158 m-PCRs were performed (Figure 1). Among them, 52 were excluded from the analysis. The main reason for exclusion was the absence of a simultaneous microbiological culture.

Table 1 summarizes the patients’ characteristics. They were mainly men (68%), with a mean age of 632 ± 11 years. Comorbidities were frequent, with 41% of the patients suffering from diabetes and 43% from arterial hypertension. Almost three-quarters of our patients were suffering from SARS-CoV-2 infection. Suspected diagnosis was a CAP in 15%, a HAP in 39%, and a VAP in 46% of cases. The death rate in the ICU was 54%.

### 2.2. Microbiological Data

Clinical samples were 85 ETA (82%) and 19 BAL (18%). The mean time to obtain m-PCR results was 17.7 ± 14.6 h. The PCR and culture were positive, respectively, in 55% and 38% of cases. Table 2 summarizes the results of the PCR and conventional culture. *Staphylococcus aureus* was the most frequently detected microorganism by m-PCR in 21% of cases, followed by *Haemophilus influenzae* in 13% of cases and *Escherichia coli* in 11% of cases. *S. aureus*, *Enterobacter aerogenes*, and *Pseudomonas aeruginosa* were the bacteria most often isolated by conventional culture, respectively, in 8%, 7%, and 6% of cases. The bacteria identified only by m-PCR (false positive) were mainly *H. influenzae* (*n* = 12/14) and *S. aureus* (*n* = 14/22). The pathogens identified only by culture (false negative) were *Hafnia alvei* (*n* = 4/4), *Aspergillus* sp. (*n* = 4/4), *Stenotrophomonas maltophilia* (2/2),and *Enterococcus faecalis* (*n* = 1/1). Regarding antibiotic resistance, the m-PCR detected 5 mechanisms of resistance, including 3 blaCTX-M, 1 blaNDM, and 1 mecA/C. These mechanisms of resistance were all confirmed by culture.

### 2.3. Performance of the m-PCR

Table 3 summarizes the analysis of concordance between the results of m-PCR and those of conventional microbiological methods. It showed a global sensitivity of 50% (IC95% [0.31, 0.69]), a specificity of 55% (IC95% [0.43, 0.67]), a PPV of 29% (IC95% [0.17,0.44]), and a NPV of 75% (IC95% [0.62, 0.86]) (Kappa = 0.06; IC95% [−0.11, 0.24]; *p* = 0.49). Regarding ETA samples, the analysis of concordance revealed a sensitivity of 57% (IC95% [0.34, 0.78]), a specificity of 53% (IC95% [0.40, 0.66]), a PPV of 29% (IC95% [0.16, 0.45]) and a NPV of 79% (IC95% [0.64, 0.90]) (Kappa = 0.08 IC95% [−0.11; 0.26]; *p* = 0.41).

### 2.4. Impact of m-PCR Results on Antibiotic Prescription and Additional Modifications after Culture Results

The impact of m-PCR results on antibiotic prescription is summarized in Table 4. In the case of a negative m-PCR, an appropriate antibiotic strategy was applied in 16 (34%) cases, with a lack of antibiotic initiation in 8 cases and an antibiotic interruption in 8 cases. Regarding positive m-PCRs, 51 (89%) led to an appropriate antibiotic prescription, including 33 appropriate initiations, 9 escalations, 7 de-escalations, and 2 optimizations. In 6 cases, antibiotherapy following positive m-PCR results was inappropriate. Three *S. aureus* pneumonia and one polymicrobial pneumonia (*H. influenzae*, *S. aureus*, *M. catarrhalis*) were treated with piperacillin–tazobactam without de-escalation. Amoxicillin–clavulanic acid was continued in one case despite the detection of gene mec A/C and in one episode of polymicrobial pneumonia despite the detection of *P. aeruginosa*.

**Figure 2:**
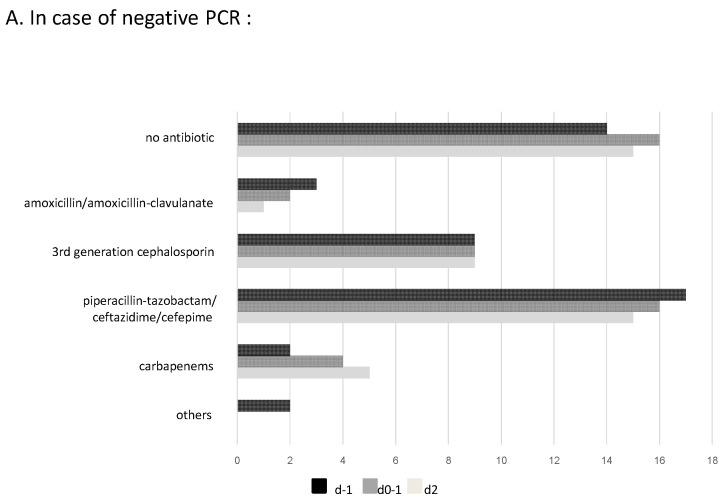

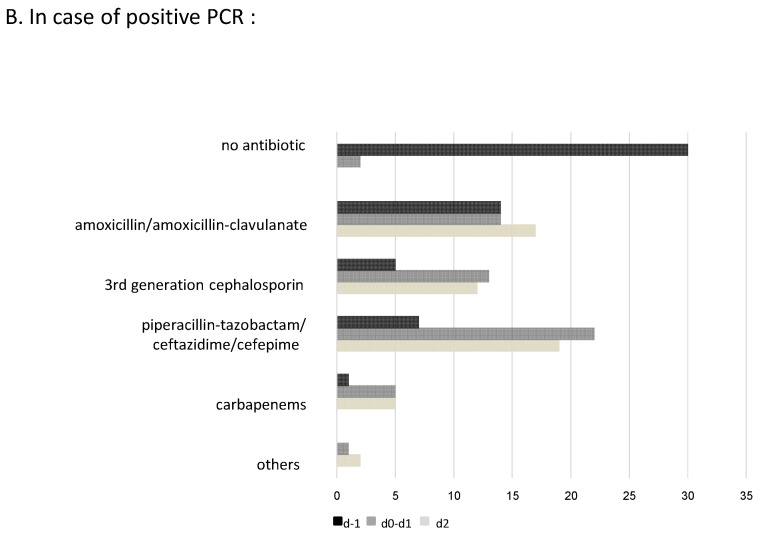
Antibiotic prescriptions after m-PCR and conventional culture results: (**A**) In case of negative PCR and (**B**) In case of positive PCR.

Figure 2 shows initial antibiotic prescriptions, changes following m-PCR results, and additional modifications after culture results. Four patients died between day 1 and day 2 after the onset of pneumonia, preventing antibiotic change depending on microbiological culture results. On day 2, in cases of a negative m-PCR, obtaining the results of culture and antibiogram never led to the cessation of antibiotics, despite a negative culture in 75.4% of cases. In cases of positive m-PCR, antibiotic therapy was modified after cultureresults in 17.5% of cases. In 4 cases, antibiotic change was motivated by resistance to initial probabilistic treatment. In 2 cases, antibiotic escalation was applied despite bacterial sensitivity to ongoing treatment. In 4 cases, negative standard culture motivated antibiotic de-escalation.

### 2.5. Factors Associated with Appropriate Antibiotic Strategy after m-PCR Results

Table 5 summarizes the univariate and multivariate analysis of factors associated with an appropriate antibiotic strategy following m-PCR results. In univariate analysis, factors associated with appropriate antibiotic strategy following m-PCR results were history of chronic respiratory insufficiency (*p* = 0.05), lower SAPSII score (*p* = 0.03), absence of prior antimicrobial therapy within one month (*p* < 0.01), and a positive m-PCR (*p* < 0.01). In multivariate analysis, identification of at least one bacteria by m-PCR (OR: 96.60; IC95% [9.72, 960.20], *p* < 0.001) and a lower SAPSII score (OR: 0.96; IC95% [0.931, 0.997], *p* = 0.034) were significantly associated with an early appropriate change ininitial probabilistic antibiotic therapy following the results of m-PCR. 

## 3. Discussion

This study aimed to evaluate the impact of m-PCR on the real-life antibiotic management of mechanically ventilated patients suspected of pneumonia in a polyvalent intensive care unit. It demonstrated an impact of m-PCR on antibiotic strategy in nearly twothirds of cases. A positive m-PCR result allowed for the initiation or early adjustment of antibiotic therapy in almost 90% of cases. A negative m-PCR result spared antibiotic use in over onethird of cases.

In our study, m-PCR results were compared withthose of standard culture obtained 48 to 72 h after sample collection. We found a sensitivity of 50% and a specificity of 55%. The positive and negative predictive values were 29% and 75%, respectively. The concordance between m-PCR and culture was 55%, consistent with the concordance of 56% reported by Crémet et al. in a similar population [18]. The concordance improved with the bacterial load. A >90% concordance was reported for culture values above 10^6^/mL [19]. Higher performances have been reported in similar studies in patients with and without COVID-19,with sensitivities and specificities exceeding 80% [20,21,22]. Our study revealed that m-PCR led to the identification of additional bacteria compared withstandard culture. Accordingly, Monard et al.found nearly twice as much microbiological documentation with m-PCR compared with culture [21]. Buchan et al. demonstrated a 94.8% increase in thenumber of detected bacteria, while Lee et al. reported a 70.3% increase [21,23].

The false positive results of m-PCR mostly revealed *H. influenzae* and *S. aureus*, as reported by others [23,24]. Crémet et al. showed that, for over 50% of m-PCR positive for *H. influenzae*, either the culture turned positive after using an enriched medium, or this bacterium was finally overgrown in culture by other pathogens from the commensal flora [18]. *S. aureus*, *H. influenzae*, *M. catarrhalis*, and *P. aeruginosa* were the bacteria most frequently associated with false-positive m-PCR results. Murphy et al. found similar results on 845 BAL and 846 sputum and ETA [19]. Standard culture has limitations since the culturing techniques are based on the detection of dominant pathogens. Minor bacteria and exigent pathogens such as *H. influenzae* can be missed. Given the retrospective nature of our study and its real-life conditions, no additional microbiological tests were performed in case of discordant results. The concordance between m-PCR and culture is also influenced by prior antibiotic use, which can produce negative cultures. Our results showed that 47% of false-positive m-PCR results involved patients who had received at least one dose of an antibiotic active against the identified bacterium within the 24 h preceding the sampling. Similar findings were reported by Buchan et al., with 49% of false positive m-PCR being from patients who had received antibiotics within 72 h [20]. Taking into account bacteria isolated in culture at rates below the threshold of significance also reduces the discordance between the two techniques [16,17]. The FilmArray^®^ pulmonary panel provides semi-quantitative results expressed in copies/mL ranging from 10^4^ to ≥10^7^ copies/mL. Within this range, the concordance between m-PCR and culture is accurate within a 0.5 log difference [19,25]. The culture of ETA samples often shows negative results for PCR-positive samples at 10^4^ copies/mL. This raises the question of the contribution of m-PCR in patient management when positive at a low concentration with a negative culture, suggesting potential contamination of the sample with oral flora. Concordance is also improved when the results of repeated cultures surrounding m-PCR are taken into account [18,26]. In our population, the majority of patients underwent ETA rather than bronchoalveolar lavage (BAL), increasing the m-PCR false positive results. The m-PCR in ETA showed a sensitivity of 57%, a specificity of 53%, a positive predictive value of 29%, and a negative predictive value of 79%. This high negative predictive value could encourage antibiotic de-escalation in cases of negative m-PCR in ETA samples [27]. We described 14 false negative m-PCR results involving *H. alvei*, *Aspergillus* sp., and *S. maltophilia* that are not included in the m-PCR panel. These results confirm that m-PCR should not be performed alone, as culture remains necessary to detect m-PCR false negativesand to perform antimicrobial susceptibility testing.

The real-life impact of the m-PCR in our ICU was evaluated by the proportion of antibiotic strategies aligned with the m-PCR results. For positive m-PCR results, 89% of patients had appropriate antibiotic strategies, including 58% initiations, 16% escalations, and 12% de-escalations. On day 2, antibiotic therapy was modified based on standard culture results in only 17.5% of cases. For negative m-PCR results, the absence of antibiotic initiation or discontinuation occurred in over onethird of our patients. Previous studies have demonstrated the potential of m-PCR in reducing unnecessary antibiotic treatment, reporting significant reductions in the duration and overall use of antibiotics. Most of these studies simulated the impact of m-PCR results by comparing the antibiotics prescribed in practice by clinicians unaware of the m-PCR results to those chosen in theory by experts informed of the m-PCR results. The anticipated proportion of antibiotic de-escalation when using m-PCR was around 40% in the literature [16,21,28,29,30]. For instance, Guillotin et al. showed only 37% of predicted broad-spectrum antibiotic therapies when using m-PCR compared with 88% when following clinical guidelines [28]. Buchan et al. reported a potential de-escalation or discontinuation of antibiotic therapy based on m-PCR results in 48% of patients, resulting in an average saving of 6.2 antibiotic days/patient. Two prospective randomized studies evaluated the impact of m-PCR on antibiotic use [20]. Darie et al. found a reduction in the duration of inappropriate antibiotic therapy by 38.6 h [31]. However, the conclusions of this study were limited by the mild severity of the patients and the low rate of bacterial documentation [32]. Farthouk et al. assessed the impact of m-PCR coupled with PCT. They did not demonstrate a significant reduction in antibiotic use, although it suggested a possible antibiotic-sparing effect [33].

The proportion of antibiotic de-escalation when using m-PCR was only 12% in our real-life cohort. Our results were comparable to those reported by Maataoui et al., who found 11% de-escalation after m-PCR results in cases of SARS-CoV-2-related pneumonia in a retrospective cohort [17]. Similarly, the DIANA study, which evaluatedantibiotic de-escalation in infected intensive care unit patients, found 16% de-escalation [34]. No deleterious impact of de-escalation was observed on clinical recovery. Tabah et al. reported that de-escalation was more often applied in patients with an already favorable clinical course [30].

A negative m-PCR led to antibiotic sparing by discontinuation or absence of antibiotic initiation in 34% of our patients. Maataoui et al. and Posteraro B et al. reported similar results in patients suffering from COVID-19 [17,32]. In our cohort, a high SAPSII score was significantly associated with the lack of consideration for m-PCR results. A negative culture confirming the m-PCR result did not lead to additional discontinuation of antibiotics on days 2 and 3, highlighting the reluctance of physicians to discontinue antibiotics in the most criticallyill patients. Maataoui et al. also found that patient severity encouraged the continuation of antibiotics in half of the cases for at least 48 h, despite the high NPV of a negative m-PCR [17]. Conversely, a negative m-PCR in patients with minor symptoms could safely result in the absence of antibiotic therapy. The algorithm proposed by Novy et al. in cases of a negative m-PCR showed antibiotic sparing in 65% of samples. They suggested discontinuing empirical antibiotic therapy if the m-PCR is negative and the patient does not present any severity criteria, such as septic shock or ARDS, with no Gram-negative bacteria observed on direct examination [35]. This strategy requires considering the local ecological risk in each ICU.

Our study has several limitations. We could not include all m-PCRperformedwhen concomitant cultures were lacking. The study was monocentric, which prevents the extension of results to other centers with different ecologies and antibiotic strategies. Due to the observational approach, factors related to the physicians, patients, and type of infection were not controlled. The suspicion of pneumonia remained at the discretion of the attending physician. The presence of other infectious foci indicating the continuation of antibiotic therapy wasnot collected, nor were clinical criteria such as clinical improvement. Samples were sometimes collected on weekends without a microbiology team available to provide rapid results, explaining the large variation in the turnaround time for m-PCR results. Of note, 74% of the patients included in our study had SARS-CoV-2 infection. Due to the severity of the patients, their high mortality rate, and the initial lack of data on the incidence of bacterial superinfections, the proportion of empirical antibiotic therapy was high, and de-escalation criteria were limited.

In conclusion, our study demonstrates the potential of multiplex pulmonary PCRin improving the appropriateness of empirical antibiotic therapy and reducing antibiotic use in cases of suspected nosocomial or VAP, impacting real-life clinical practice. However, further researchisneeded to define better which patients will benefit the most from m-PCR use and to assess its impact on clinical outcomes. Overall, multiplex PCR holds promise as a valuable tool for rationalizing antibiotic therapyin respiratory infections.

## 4. Patients and Methods

### 4.1. Setting and Study Population

We conducted an observational monocentric retrospective study in Victor Provo Hospital (Roubaix, France), which is a general hospital of 750 beds with a medical and surgical ICU of 25 beds. Between November 2020 and March 2022, ventilated patients with suspected pneumonia hospitalized in our ICU who benefited from respiratory sampling simultaneously tested using conventional microbiological methods and m-PCR (Film Array^®^ Panel Pneumo Plus, bioMérieux^®^, Marcy l’Etoile, France) were included. Patients who were not ventilated at the onset of pneumonia and benefited from an m-PCR performed on a sputum sample and those who died within 24 h of the onset of pneumonia were excluded from the analysis. The m-PCR was performed at the physician’s request in the bacteriology laboratory. Cases were identified using a laboratory database query completed with the ICU clinical databases analysis. Patients who had multiple episodes of suspected pneumonia could be included several times. 

### 4.2. Microbiological Analysis

Respiratory specimens were routinely analyzed using conventional microbiological methods (gold standard). Identification and in vitro antimicrobial susceptibility testing were performed with the Vitek 2 system (bioMérieux^®^, Marcy l’Etoile, France) according to the European Committee on Antimicrobial Susceptibility Testing (EUCAST) breakpoints [36]. Results of a standard culture were expressed in colony-forming unit (CFU)/mL. The thresholds for positivity on culture were ≥10^4^ CFU/mL for bronchoalveolar lavages (BAL) and ≥10^5^ CFU/mL for endotracheal aspirations (ETA). Respiratory samples were simultaneously tested usingthe m-PCR (Film Array^®^ Panel Pneumo Plus). The system integrates sample preparation, nucleic acid extraction and purification, amplification, detection, and analysis with a total run time of about 1 h. This test enables rapid and accurate detection of 18 bacteria (*Acinetobacter calcoaceticus-baumanii*, *Enterobacter cloacae*, *Escherichia coli*, *Haemophilus influenzae*, *Klebsiella aerogenes*, *Klebsiella oxytoca*, *Klebsiella pneumoniae*, *Moraxella catarrhalis*, *Proteus* sp., *Pseudomonas aeruginosa*, *Serratia marcescens*, *Staphylococcus aureus*, *Streptococcus agalactiae*, *Streptococcus pneumoniae*, *Streptococcus pyogenes*, *Chlamydia pneumoniae*, *Legionella pneumophila*, *Mycoplasma pneumoniae*), sevenantibiotic resistance markers (mecA/C and MREJ, IMP, KPC, NDM, OXA-48-like, VIM, CTX-M), and nine viruses (Adenovirus, Coronavirus, Rhinovirus/Enterovirus, Metapneumovirus, Influenza A and B, Parainfluenza virus, Respiratory syncytial virus, Middle East respiratory syndrome coronavirus). We only analyzed bacteria and antibiotic resistance detection. The results are expressed as semi-quantitative results (10^4^ to ≥10^7^) in DNAcopies/mL for identified bacteria and as qualitative results (presence or absence) for resistance genes. 

### 4.3. Data Collection 

Clinical and demographical data were retrospectively obtained from the medical files of each patient. The following data were recorded on ICU admission: demographic characteristics (age, gender), indication(s) of ICU admission, underlying clinical conditions, immunodeficiency, and severity of illness at admission. At the time of pneumonia diagnosis, classification of pneumonia (ventilator-associated pneumonia, VAP; hospital-acquired pneumonia, HAP; community-acquired pneumonia, CAP), prior antimicrobial therapy within one month, duration of hospital and ICU stay, and severity of illness were collected. Antimicrobial prescriptions were recorded on the day before and day 0, day 1, day 2, and day 3 after m-PCR performance for suspected pneumonia. Antimicrobial changes after the results of m-PCR (d0–d1) and after the results of culture and susceptibility testing (d2–d3) were analyzed. All patients were follow-up until death or release from the ICU. 

### 4.4. Endpoints

The main judgment criterion was the proportion of appropriate changes in initial probabilistic antibiotic therapy following m-PCR results. An appropriate change was defined as a lack of initiation or interruption of antibiotic therapy in the case of a negative m-PCR result; an appropriate initiation, escalation, optimization, or de-escalation of antibiotic therapy in the case of a positive m-PCR result. An appropriate initiation corresponded to the introduction of an effective antibiotic on the bacteria identified by m-PCR, not treated by probabilistic antibiotic therapy preceding the m-PCR result. Optimization was defined as the use of a 4th generation cephalosporin in place of a 3rd generation cephalosporin in case of detection of group 3 enterobacteria. De-escalation included switching from one beta-lactam to another one with a narrower spectrum and lighter selective pressure according to a six-rank consensual classification of beta-lactams [37]. The proportion of inappropriate changes is defined by the rate of sampling for which the m-PCR result was not taken into account in the antibiotic strategy.

The secondary objectives of our study were to determine the factors significantly associated with an appropriate change in initial probabilistic antibiotic therapy following the results of them-PCRanalysis and to assess the concordance between the results of pulmonary m-PCR and those of conventional microbiological tests (gold standard). Detection by m-PCR of a bacteria not present in culture was considered false positive, and the absence on m-PCR of a bacteria present in culture was considered false negative. Cases where the cultural test detected a pathogen that could not be identified by the m-PCR were considered false negatives. Concordance was defined by complete qualitative agreement between m-PCR and conventional culture results. We particularly assessed the performance of m-PCR in the subcategory of ETA sampling.

### 4.5. Statistical Analysis

Continuous variables were expressed as mean values ± standard deviation or as median (interquartile range), depending on the normality of their distribution. They were compared using the Student’s test or the Mann–Whitney U test, as appropriate. Categorical variables were expressed as percentages and evaluated using the chi-square test and Fisher’s test when appropriate. Differences between groups were considered to be significant for variables yielding a *p*-value ≤ 0.05. To determine the independent effect of the variables on the appropriate change ininitial probabilistic antibiotic therapy following the results of m-PCR, we performed a logistic regression analysis using the purposeful selection of covariates. PaO_2_/FiO_2_ ratio, COVID-19 status, duration of mechanical ventilation, Simplified Acute Physiology Score (SAPS) II score, antibiotic treatment at the time of diagnosis of pneumonia, and all covariates with *p* < 0.2 in the unadjusted model were entered into the multivariate model. To assess the concordance between the results of m-PCR and those of conventional microbiological culture (reference method), the calculation of sensibility, specificity, positive predictive value (PPV), and negative predictive value (VPN) was performed, and a concordance analysiswas conducted usingthe Cohen Kappa test globally and in the group of patients who benefited from tracheal aspiration. All statistical analyses were performed using R-software^®^, version 4.3.2. 

## Figures and Tables

**Table 1:** Patients characteristics.

Patients Characteristics	Data
Demographic characteristics	
Sexe (male)	71 (68%)
Age (years)	62 ± 11
Comorbidities	
diabetes	43 (41%)
arterial hypertension	46 (43%)
respiratory chronic insufficiency	24 (23%)
renal chronic insufficiency	10 (9%)
cancer	5 (5%)
hemopathy	16 (15%)
immunodepression	22 (21%)
Clinical characteristics on the day of the PCR test	
SARS-CoV-2 infection	76 (73%)
SAPS II	49 ± 21
receiving antibiotics	90 (87%)
septic shock	71 (67%)
PaO_2_/FiO_2_	150 ± 69
duration of mechanical ventilation (days)	5.5 ± 7.2
Microbiological diagnostic	
ETA	85 (82%)
BAL	19 (18%)
delay of PCR results (hours)	17.8 ± 14.6
positive PCR	57 (55%)
positive culture	39 (38%)
Outcome	
total duration of stay in ICU (days)	35 ± 39
Deaths in ICU	56 (54%)

Mean ± standard deviation, number (%). ETA: endotracheal aspiration, BAL: bronchoalveolar lavage.

**Table 2:** Microbiological data.

Microorganisms	Identified by PCR *n* = 104	Isolated by Culture *n* = 104
Gram-positive		
*S. aureus*	22 (21%)	8 (8%)
*S. pneumoniae*	5 (5%)	2 (2%)
*S. agalactiae*	1 (1%)	0
*E. faecalis*	0	1 (1%)
Gram-negative		
*H. influenzae*	14 (13%)	1 (1%)
*E. coli*	12 (12%)	6 (6%)
*E. aerogenes*	10 (10%)	7 (7%)
*P. Aeruginosa*	9 (9%)	6 (6%)
*K. Pneumoniae*	6 (6%)	5 (5%)
*M. catarrhalis*	4 (4%)	0
*Proteus* spp.	4 (4%)	2 (2%)
*E. cloacae*	3 (3%)	2 (2%)
*S. marcescens*	1 (1%)	0
*K. oxytoca*	1 (1%)	1 (1%)
*H. alvei*	0	4 (4%)
*S. maltophilia*	0	2 (2%)
*M. morganii*	0	1 (1%)
Fungi		
*Aspergillus* spp.	0	4 (4%)

Number (%).

**Table 3:** Culture and m-PCR table of concordance in all samples and in ETA only.

	All Cultures (ETA and BAL)			
All m-PCR (ETA and BAL)		positive	negative	
	positive	14	34	48
	negative	14	42	56
		28	76	104
		Cultures in ETA		
m-PCR in ETA		positive	negative	
	positive	12	30	42
	negative	9	34	43
		21	64	85

ETA: endotracheal aspiration, BAL: bronchoalveolar lavage.

**Table 4:** Proportion of appropriate antibiotic strategies following m-PCR results.

Results of PCR *n* = 104	Appropriateness of Antibiotic Strategy	Number (%)
Negative PCR *n* = 47	Appropriate strategy	16/47 (34%)
	Antibiotics discontinuation	8/47 (17%)
	Lack of antibiotic initiation	8/47 (17%)
Positive PCR *n* = 57	Appropriate strategy	51/57 (89%)
	Appropriate initiation	33/57 (58%)
	Appropriate escalation	9/57 (16%)
	Appropriate de-escalation	7/57 (12%)
	Optimization	2/57 (4%)

**Table 5:** Univariate and multivariate analysis of factors associated with an appropriate antibiotic strategy following m-PCR results.

Variables	Factors Associated with an Appropriate Antibiotic Strategy			
	Univariate		Multivariate	
	OR [CI95%]	*p*	OR [CI95%]	*p*
Sexe (Men)	2.43 [1.05–5.70]	0.1	1.29 [0.31–6.27]	0.72
Age	1.00 [0.96–1.04]	0.93		
BMI	0.98 [0.91–1.06]	0.67		
Diabetes	0.55 [0.24–1.22]	0.18	0.69 [0.18–2.62]	0.58
Arterial hypertension	0.92 [0.41–2.06]	0.84		
Chronic respiratory insufficiency	3.54 [1.21–13.01]	0.05	3.73 [0.74–22.79]	0.12
Chronic renal insufficiency	5.64 [1.00–106.22]	0.09	9.93 [0.84–303.52]	0.10
Cancer	0.83 [0.13–0.85]	1		
Hemopathy	0.32 [0.11–0.92]	0.11	0.18 [0.01–2.31]	0.19
Immunodepression	0.33 [0.13–0.85]	0.07	0.59 [0.07–4.66]	0.62
Antibiotic allergy	0.55 [0.06–4.70]	0.61		
COVID +	0.80 [0.31–1.96]	0.83		
Type of pneumonia				
CAP	Ref.	0.3		
VAP	2.33 [0.73–7.53]			
HAP	1.67 [0.96–1.00]			
SAPS II	0.98 [0.96–1.00]	0.03	0.96 [0.93–1.00]	0.035 *
Septic shock	0.75 [0.31–1.74]	0.68		
Receiving antibiotics	0.23 [0.08–0.56]	<0.01	0.38 [0.09–1.47]	0.17
PaO_2_/FiO_2_	1.0 [0.99–1.00]	0.58	1.00 [0.99–1.02]	0.34
Sampling				
ETA	Ref.	0.89		
BAL	1.15 [0.43–3.31]			
Positive m-PCR	17.33 [6.52–63.09]	<0.01	107.4 [16.04–1727.65]	<0.01 *
Ventilation duration	1.03 [0.97–1.10]	0.34	1.02 [0.93–1.13]	0.69

* Statistically significant values. BMI: body mass index, CAP: community-acquired pneumonia, VAP: ventilator-associated pneumonia, HAP: hospital-acquired pneumonia, ETA: endotracheal aspiration, BAL: bronchoalveolar lavage. Ref.: reference.

## Data Availability

Data are available under request by email to the corresponding author.
